# Early Warning Method for Public Health Emergency Under Artificial Neural Network in the Context of Deep Learning

**DOI:** 10.3389/fpsyg.2021.594031

**Published:** 2021-02-15

**Authors:** Shuang Zheng, Xiaomei Hu

**Affiliations:** ^1^College of Media and International Culture, Zhejiang University, Hangzhou, China; ^2^School of Media and Law, NingboTech University, Ningbo, China

**Keywords:** public health emergencies, artificial neural network, convolutional neural network, structural equation model, early warning

## Abstract

The purpose is to minimize the substantial losses caused by public health emergencies to people’s health and daily life and the national economy. The tuberculosis data from June 2017 to 2019 in a city are collected. The Structural Equation Model (SEM) is constructed to determine the relationship between hidden and explicit variables by determining the relevant indicators and parameter estimation. The prediction model based on Artificial Neural Network (ANN) and Convolutional Neural Network (CNN) is constructed. The method’s effectiveness is verified by comparing the prediction model’s loss value and accuracy in training and testing. Meanwhile, 50 pieces of actual cases are tested, and the warning level is determined according to the *T*-value. The results show that comparing and analyzing ANN, CNN, and the hybrid network of ANN and CNN, the hybrid network’s accuracy (95.1%) is higher than the other two algorithms, 89.1 and 90.1%. Also, the hybrid network has sound prediction effects and accuracy when predicting actual cases. Therefore, the early warning method based on ANN in deep learning has better performance in public health emergencies’ early warning, which is significant for improving early warning capabilities.

## Introduction

With the continuous development of the economy, culture, and technology of all countries globally, people’s life quality is gradually improving. However, due to society’s rapid development, the ecological environment has brought more uncertainty to human beings’ survival status. Emergencies have also become a norm for various countries. Many countries can actively respond to unexpected economic and technological situations through improvement and innovation. However, health emergencies may cause substantial losses worldwide (Xu et al., 2017; Carleton et al., 2019). For example, the white anthrax powder incident in the United States has brought the international community into an emergency state in response to public health incidents. Subsequent Severe Acute Respiratory Syndrome (SARS) incident, highly pathogenic avian influenza, Streptococcus suis infection, hand-foot-mouth disease outbreaks, Ebola, and Corona Virus Disease 2019 (COVID-19) pandemic at the end of 2019 have all sounded alarm bells one after another for the global public health safety issues (Carleton et al., 2018; Stroeymeyt et al., 2018).

After the infectious diseases in the past were detected, various countries have actively taken countermeasures to control them well. The SARS incident in 2003 also started the emergency management of major public safety incidents in China. China continually reflects on the management of public crisis events. While governments at all levels continue to strengthen economic regulation and market supervision, they should value the social management and social public service functions so that they can respond quickly and effectively to emergencies and risks (He et al., 2020; Zhou et al., 2020). Simultaneously, after the SARS incident, China also wrote an “emergency” to the Constitution. Afterward, the social early warning system and emergency response mechanism are gradually established and improved as much as possible to improve the ability to handle emergencies and protect people’s lives and property safety. The COVID-19 has also spread throughout the world. This major pandemic has also caused severe threats to human health and impacts people’s daily lives, social development, the market economy, and national security (Piciullo et al., 2018; Shenfield et al., 2018; Syafrudin et al., 2019).

The deep learning technology’s continuous development provides possibilities for many fields. Deep learning can solve classification problems. Neural networks and Long Short-Term Memory (LSTM) networks can solve text recognition problems. Especially, there are numerous information sources in the big data context. How to transform public information data into adequate support to improve information and countermeasure management has become an essential problem to be solved by information technology (Carleo and Troyer, 2017; Annarumma et al., 2019).

In summary, public health emergencies’ early warning is significant for people all over the world. Therefore, in deep learning, the Artificial Neural Network (ANN) constructs an early warning system for public health emergencies. The relevant news and other information data are intelligently extracted and analyzed to predict the early warning level of events, assisting relevant departments to improve the emergency detection efficiency. It guarantees the follow-up management and coordination work, thereby reducing all kinds of losses caused by public health emergencies to society.

## Literature Review

In recent years, there have been more or less public health incidents in countries around the world. Experts and scholars are actively exploring early warning systems to reduce the losses in various aspects as much as possible when public health events occurred, thereby reducing public health events’ impact on people. Huang and Xiang (2018) proposed a deep belief network method based on the Softmax classifier and Dropout mechanism to reduce rainfall-induced landslide disasters. Its powerful non-linear mapping ability was used to extract the landslide factors’ inherent characteristics. The algorithm’s advantages in accuracy and technology were verified by practical cases (Huang and Xiang, 2018). Yang et al. (2020) predicted high-risk students using the Convolutional Neural Network (CNN)’s learning image recognition function. The results showed that the two proposed methods could perform better than algorithms such as support vector machines, random forests, and deep neural networks. Its average recall rate reached 77.26%, indicating this method’s effectiveness (Yang et al., 2020).

To detect blood infections early, identify the type of pathogens, and treat them in time, Van Steenkiste et al. (2018) applied long-term memory networks to predict the results of blood culture experiments. The prediction results were also relatively accurate (Van Steenkiste et al., 2018). Jang et al. (2020) used ANNs to develop and test their classifiers. The test results were used to predict cardiac arrest in the emergency department. Also, the dangerous patients of cardiac arrest were predicted by training multi-layer perceptron, long-term memory, and mixed memory. The results indicated that the dangerous patient would have a cardiac arrest 24 h after the prediction, showing the prediction model’s excellent performances (Jang et al., 2020). Guo et al. (2020) established an early warning model using ANNs to accurately grasp disease prevention and treatment timing. By analyzing concentrated infectious diseases in China, the epidemic intensity and the need to issue early warning signals were comprehensively determined. The experimental results also validated the method’s effectiveness in determining epidemic conditions (Guo et al., 2020).

Most of the past investigations detect and predict public emergencies through the current data situation and purpose. For some public health emergencies, data collection and analysis are significant. Emergency management efficiency is currently low. ANN builds an early warning model with significant influences using deep learning to warn people by analyzing intelligence data due to public health emergencies.

## Materials and Methods

### Artificial Neural Network

ANN is a simulated human nervous system, and information is output and transmitted through countless neurons. The human brain can process complex information. The reason is the countless neurons in the human nervous system, and they are non-linear in processing the input information. Therefore, the computer can imitate the human brain’s thinking process to help solve practical problems (Li H. et al., 2017; Mocanu et al., 2018; Zador, 2019).

**FIGURE 1:**
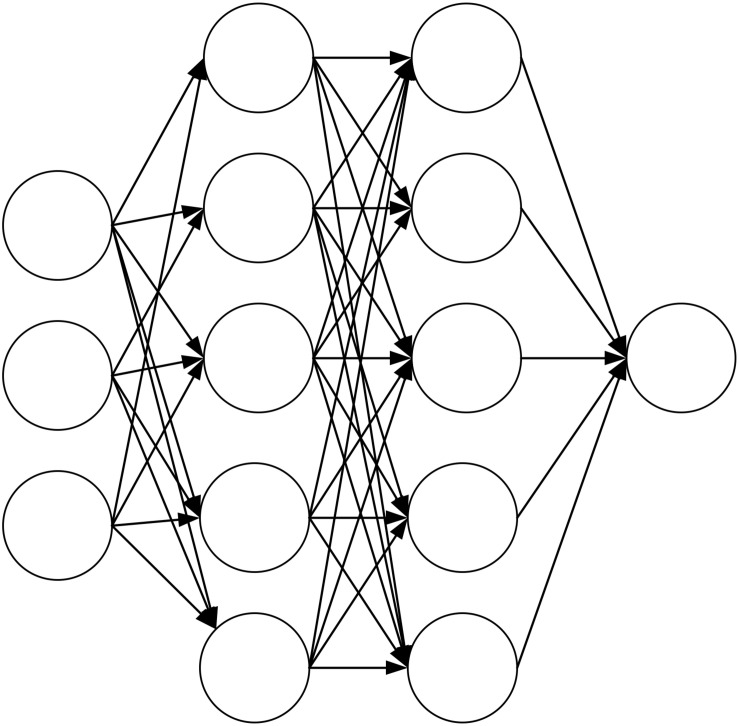
The structure diagram of ANN.

The most widely used ANN is the three-layer neural network structure. The structure includes an input layer, a hidden layer, and an output layer. The input layer accepts data. The hidden layer processes and converts input signal data and determines the output signal. The output layer transfers the network’s processing results. The input layer determines input nodes based on the number of input variables. According to different processing software and processing strategies, the output layer corresponds to different output nodes (Tealab, 2018; Jia et al., 2019). The ANN structure is shown in Figure 1.

Through continuous sample data training, the network weights are continuously adjusted to minimize the prediction error. When the error after each learning is considerable, it is necessary to continue learning and iterate continuously until the termination condition is satisfied. The input node has no upper node connected to it, and other nodes need to use the output of the upper node as the input value to complete the training further. It needs to go through the adder and activation function to complete the node work for each node. When *X* represents the input node’s value, and y represents the output node’s output signal, the node j’s accelerator can be defined as Eq. 1.

(1) $$U_{f}=\sum_{i=1}^{n}w_{i\,j}\,x_{i}+\theta_{j}$$

where *w*_*ij*_ represents the weight between the upper layer’s node *i* and this layer’s node j in the two adjacent network layers, θ represents the node deviation, regarded as a constant term, *n* denotes the number of nodes in the upper layer, and *x*_*i*_ denotes the output of the node *i*.

Besides, in the network, the activation function is also relatively important. It is assumed that the activation function of the node *j* can be expressed by Eq. 2.

(2) $$y_{j}=f\,\left(U_{j}\right)$$

where *y*_*j*_ represents the activation function value, i.e., the node’s output value, and *U*_*f*_ in the adder refers to the input value in the activation function.

The Sigmoid function is the most commonly used activation function in ANN (Zhou et al., 2018; Ayzel et al., 2019), described by Eq. 3.

(3) $$f\,\left(U_{j}\right)=\frac{1}{1+e^{-U_{j}}}$$

### CNN

**FIGURE 2:**
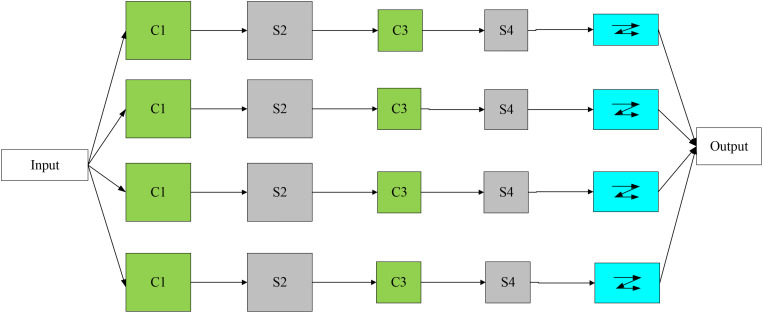
CNN structure.

CNN is a significant and widely used network model in deep learning technology. It is a supervised learning model. The network connection method is also changed from full connection to partial connection, which reduces the parameter number in the network and the training process complexity and effectively improves the analysis efficiency (Guo et al., 2018; Hussain et al., 2018; Kriegerowski et al., 2019). CNN has good performance in image recognition and processing. It can complete feature extraction through image segmentation, convolution, pooling, and other operations. Its structure is shown in Figure 2.

CNN’s structure includes an input layer, a convolutional layer (C1, C3), a pooling layer (S2, S4), a connection layer, and an output layer. Among them, the convolutional layer and the pooling layer constitute the hidden layer. Convolution and pooling operations are performed by extracting the input value’s features, and the local features are integrated into all features for output (Montoro et al., 2018; Tommasetti et al., 2018).

### Structural Equation Model

The Structural Equation Model (SEM) is a multivariate statistical technique that combines factor analysis and path analysis. It makes up for traditional statistical methods’ shortcomings and becomes an essential tool for multivariate data analysis. SEM includes the measurement model and the structural model. The structural model is the internal model, mainly representing hidden variables, that is, the causal relationship between unobservable variables. The measurement model represents the external model, representing the explicit variables: the relationship between the observable variable and the hidden variable (Jiang et al., 2018; Jia et al., 2019).

In the structural model, ellipses represent hidden variables. The causal relationship between hidden variables is realized by the arrow pointing from cause to effect. “Cause” can also be called an endogenous hidden variable, and “effect” is called an independent variable. There may be multiple dependent variables in the structural model. A dependent variable may correspond to multiple hidden variables. The complicated causal relationship between hidden variables can be expressed by Eq. 4.

(4) η=B⁢η+Γ⁢ξ+ζ

where η represents the endogenous hidden variable, *B* and *Ã* represent the coefficient matrix, *ξ* stands for the exogenous hidden variable, and ζ stands for the error term of the structural equation.

In the measurement model, the explicit variables are represented by rectangles. The obtained weight coefficient expresses the relationship between the explicit and hidden variables. The hidden variables’ number determines the number of measurement models, which can be achieved by Eqs 5 and 6).

(5) $$y=\Lambda_{y}\,\eta +\varepsilon$$

(6) $$x=\Lambda_{y}\,\xi +\delta$$

Equation (5) describes the endogenous hidden variable, where *y* represents the explicit endogenous variable, η represents the endogenous hidden variable, and ε represents the measurement error term of *y*. Equation (3) describes the exogenous hidden variable, where *x* represents the explicit exogenous variable, *ξ* represents the exogenous hidden variable, and δ represents the measurement error term of *x*. The error terms involved in the above equations must meet the following conditions: (1) the mean is 0, and the variance is constant; (2) there is no correlation sequence; (3) the error term is not related to exogenous and endogenous hidden variables; that is, ζ is not related to δ and ε.

The following steps must be followed to establish SEM (Li C. et al., 2017; Cella et al., 2019).

Step 1: The event’s relevant background is explored. The logical relationship between explicit variables is analyzed according to the event’s relevant background. The explicit variable corresponding to each hidden variable is determined through reasoning and assumptions. Then, the model structure of the variable is obtained.

Step 2: The hidden variables and explicit variables are defined. Hidden variables are unobservable variables, and explicit variables are observable variables. The relationship between them can be understood as: the hidden variable is a high-level summary of the explicit variable, and the explicit variable is the indicator representation of the hidden variable.

Step 3: SEM’s path diagram. The model structure is set to visually show the relationship between them through graphics through the definition of variables.

Step 4: Parameter estimation. The Partial Least Squares (PLS) method estimates the SEM’s parameters. This method does not require assumptions about the data when predicting events. It has a fast convergence speed and high computing power.

**FIGURE 3:**
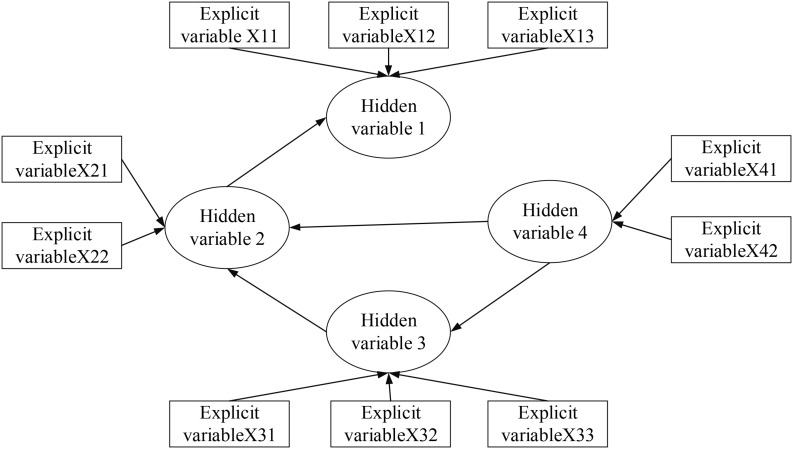
SEM path.

Therefore, the path diagram of SEM based on the above steps is shown in Figure 3. In the figure, *X1*, *X2*, *X3*, and *X4* represent hidden variables, and *X11*, *X12*, *X13*, *X21*, *X22*, *X31*, *X32*, *X33*, *X41*, and *X42* represent explicit variables.

The ANN model can express the non-linear relationship between the variables, and it also has self-learning ability. It can automatically adjust the connection weights between network nodes to fit the relationship between variables. However, its topological structure is determined by experience. Neurons are often fully connected, and the model lacks explanations for influence paths between input and output variables and the neurons. If SEM and ANN are combined, SEM can determine the causal relationship between perceptions and convert it to the ANN model’s topology. The ANN model’s non-linear mapping ability and self-learning ability can fit the causality between multiple perceptions. This method solves SEM’s linearity and difficult parameter estimation problem and establishes the well-founded ANN model topology structure. The structural Equation model can express the causal relationship between various elements and concisely show each element’s influence on the result. There are two methods for SEM: PLS and Maximum Likelihood (ML), and PLS is used adopted for structural Equation model analysis.

Therefore, a perceptual modeling method combining SEM and ANN is proposed herein. The SEM-ANN model is a structured neural network model. It can explain network nodes’ causal relationship and influence degree and improve the perceptual model’s goodness by the neural network’s non-linear fitting ability to express the relationship between perceptions accurately and quantitatively and the factors that affect perceptions. SEM can determine hidden variables and explicit variables, infer hidden variables by the explicit variables’ measurement, and test the correctness of model assumptions.

The topological structure of the SEM-ANN model is determined by the results of the SEM causal analysis. The SEM-ANN model can analyze the influence path and degree of the input variables on the output variables. The number of external measurement variables determines the number of input nodes. Assuming that there are *I* external measurement variables, then *x*_*i*_ represents the external measurement variables’ input. The numbers of hidden layers and hidden layer neurons are determined by the number of exogenous latent variables and endogenous latent variables. Assuming that there are *B* exogenous latent variables and *N* endogenous latent variables, represented by *ξ_*b*_(b = 1, 2, …, B)* and η*_*n*_(n = 1, 2, …, N)*, respectively. The number of endogenous measurement variables determines the number of output neurons. Assuming that there are *O* endogenous measurement variables, the *γ_*O*_* denotes the output of endogenous measurement variables. According to the neural network model’s topology structure, the connection weight between the input layer network node and the hidden layer neuron node is determined as a *B × I* dimensional vector. The connection weight between hidden layer neuron nodes is an *N × (B+N)* dimensional vector. The connection weight between hidden layer neuron nodes and output layer neuron nodes is an *O × N* dimensional vector. Each neuron in the neural network contains a non-linear activation function. Here, the activation function of all neurons is assumed to be the Sigmoid function.

### Data Sources and Processing

In the big data era, the information source scope continues to expand, and the information data amount has also increased explosively. Current information sources have also grown more based on traditional information sources. Traditional information sources are mainly divided into three categories. The first is the text category, such as books, magazines, government reports, brochures, and newspapers. The second is the human resources category, mainly public character interviews and related event introductions. The third is the media category, which mainly includes radio and television programs. With the continuous development of information technology and network, new information sources appear in front of the public and are divided into five categories. The first is online media, including news websites and WeChat public accounts. The second is social networks, such as WeChat, Weibo, and Dingding. The third is related literature, such as academic institutions, corporate reports, and related research papers. The fourth is the Internet of Things information, including the number of vehicles and people in public places. The fifth type of information data includes various statistical data, document databases, and archive databases. The increase in information sources brings more information data. Therefore, more sample data can be provided in the subsequent data processing to ensure that the event can be truly reflected.

In the investigation, a city’s disease control center and tuberculosis hospital are visited from June 2017 to 2019, and relevant data are grabbed from multiple information sources. A cluster of patients diagnosed with active tuberculosis was selected and reported to the China Disease Prevention and Control Information System case report card. The relevant information was derived from the China Disease Prevention and Control Information System. The tuberculosis data in all schools (high school to university) of the city from 2017 to 2019 are obtained. Cases found in physical examinations with comparatively short exposure time and complicated transmission channels are excluded; cases of school-leavers and adult education students who cannot be screened for close contacts are deleted. The comparison of many scholars’ research results shows that disease outbreaks in school three have primary causes. The first is the delay in doctors’ diagnosis. Then, there is much personnel in schools, providing conditions for disease transmission. Finally, the school dormitory’s hygienic condition is low, which cannot be ventilated and disinfected in time, providing a hotbed for diseases. Therefore, through visit surveys and data collection, ten indicators that affect the disease spread are determined, namely the patient age (PA), the students’ number in the school (SN), the school doctor ratio (SDR), the infectious exposure time (IET), the school level (SL), bacteria status case sputum (DSBS), strong positive rate of the tuberculin pure protein derivative (PPD-SPR), consecutive case number (CN), number of patients with pleurisy (NPP) in all cases, dormitory staff setting (DSS), displacement ventilation (DV), and disinfection (D-D). The units of exposure time, sputum bacteria status, dormitory staffing, students’ number, and ventilation and dormitory disinfection are not uniform. Therefore, the processing is needed to obtain the data in Table 1.

**TABLE 1:** Case data processing.

Variable	Description processing
SL	• University level is 1
	• Junior level is 2
	• Technical secondary level is 3
	• High school level is 4
NS	• The level under 5,000 people is 1
	• The level of 5,000–10,000 people (including 5,000 people) is 2
	• The level of 10,000–15,000 people (including 10,000 people) is 3
	• The level above 15,000 people is 4
SDR	Number of school doctors per 100 students
IET	• The level of cough for more than 28 days (including 28 days) is 1
	• The level of cough for 15–28 days (including 15 days) is 2
	• The level of cough for 2–14 days (including two days) is 3
	• The level of cough for less than one day (including one day) is 4
DSBS	• The negative level is 1
	• The positive level is 2
DSS	• The level for eight people is 1
	• The level for six people is 2
	• The level for four people is 3
	• The level for two people is 4
DV	• From November to January of the following year, the level is 1
	• From February to April, the level is 2
	• From May to August, the level is 3
	• From September to October, the level is 4
D-D	• Less than once a month, the level is 1
	• 1–2 times a month, the level is 2
	• 3–4 times a month, the level is 3
	• More than four times a month, the level is 4

On this basis, the indicators are statistically described, and the results in Table 2 are obtained.

**TABLE 2:** Descriptive statistics.

Indicator	Number of samples	Maximum value	Minimum value	Average	Median
PA	410	30.00	10.00	18.653	21.000
NS	410	4.00	1.00	2.594	2.000
SDR	410	1.00	0.29	5.58E -01	5.789E -02
IET	410	4.00	1.00	2.4319	3.000
SL	410	4.00	1.00	1.5142	1.000
DSBS	410	3.00	1.00	1.8896	2.000
PPD-SPR	410	4.00	0.00	0.1805	0.1500
CN	410	4.00	1.00	3.0081	1.4087
NPP	410	4.00	1.00	0.1671	0.0000
DSS	410	4.00	1.00	2.9757	3.0000
DV	410	4.00	1.00	2.1197	2.0001
D-D	410	4.00	1.00	2.3157	2.0000

The SEM of tuberculosis early warning includes five hidden variables, infection status, natural status, school status, disease status, and prevention status based on the consult to tuberculosis experts and previous scholars’ research. The infectious disease situation has two explicit variables, the natural state one, the school state three. The disease situation has three latent variables, and the prevention state has two explicit variables.

### Variable Definition and Parameter Estimates

According to the classification level of public health emergencies in China, Level I (especially serious-red), Level II (severe-orange), Level III (heavier-yellow), and Level IV (general-blue), Equation 7 is used for calculation in early warning.

(7) T=0.9⁢a+0.6⁢b+0.3⁢c+0⁢d

Where: *a*,*b*,*c*,*d* represent the event numbers of the four corresponding levels, and the corresponding coefficients are the weights given to the level events. The threat degree and early warning value range are shown in Table 3.

**TABLE 3:** Threat degree and early warning value range.

Security level	Weights	Early warning value range
I	0.9	21.3 < *T*
II	0.6	16.6 < *T*≤21.3
III	0.3	11.7 < *T* < 16.5
IV	0.0	*T*≤11.7

**FIGURE 4:**
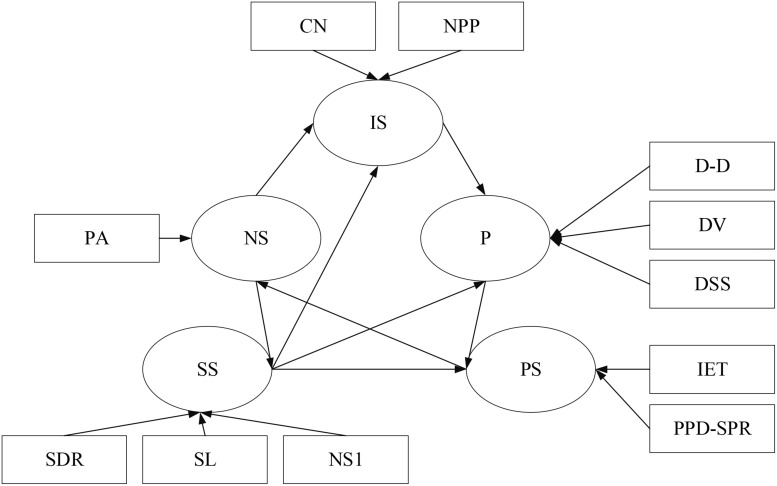
Variable path diagram.

Therefore, based on the existing literature, the infection speed (IS), natural state (NS), school state (SS), patient situation (PS), and prevention (P) are used as hidden variables. There are two explicit variables in infection speed, namely CN and NPP. There is an explicit variable in the natural state, namely PA. There are three explicit variables in the school state, namely SDR, SL, and NS1. The patient situation includes two explicit variables, namely IET and PPD-SPR. The prevention includes three explicit variables, namely DSS, DV, and D-D. According to the above variable relationship, the SEM path diagram can be obtained, as shown in Figure 4.

### Prediction Model Based on ANN and CNN

When using ANN and CNN to build a prediction model based on the SEM, two stages are involved: training learning and recognition. In the training learning stage, CNN performs supervised learning training on the sample data obtained. The input neuron’s activation value reaches the output layer via the hidden layer, and the output layer neurons respond to the network according to the input pattern. When the prediction error is large, the learning training is repeated until the termination condition is met, and the output value at this time is accurate. Then comes the recognition stage. At this stage, ANN mainly classifies and decides the information input later based on the trained network model. In this case, binary is used for output.

The neuron number in the hidden layer can be obtained by Eq. (8).

(8) $$n=2\,x_{n}+1$$

where *n* represents the neuron number in the hidden layer, and *x*_*n*_ represents the input layer’s neuron number.

The experiment evaluates the prediction model’s performances in precision, recall, and FScore. The precision, recall, and FScore are expressed by Eqs (9)–(11).

(9) $$P=\frac{p_{T\,u\,r\,e}\,\left(m_{i}\right)}{p_{T\,u\,r\,e}\,\left(m_{i}\right)+p_{F\,a\,l\,s\,e}\,\left(m_{i}\right)}$$

(10) $$R=\frac{p_{T\,u\,r\,e}\,\left(m_{i}\right)}{A\,\left(m_{i}\right)}$$

(11) $$F=\frac{2\,P\,R}{P+R}$$

where *P*, *R*, and *F* represent the precision, recall, and F-Score, respectively, *p*_*Ture*_(*m*_*i*_) denotes the true prediction number as *m*_*i*_, *p*_*False*_(*m*_*i*_) refers to the false prediction number as *m*_*i*_, and *A*(*m*_*i*_) indicates the actual *m*_*i*_ number.

The model’s accuracy reflects the model’s fitting ability, while recall is an evaluation indicator for the original sample. There are sometimes contradictions between P and R indicators. At this time, comprehensive consideration is required. The most common method is the F-Score. The F value is an evaluation indicator that integrates the P and R and can comprehensively reveal the whole.

### Experimental Setup

This experiment uses the Scrapy crawler framework to capture relevant data about tuberculosis. Also, the word segmentation and cleaning are performed. It is tested on Lenovo Intel(R) Core(TM) i5-7400CPU, 8GB running memory, windows10 operating system. The initial weight is set to a random number between (0,1) to ensure the anisotropy of subsequent weight adjustment. The learning rate is 0.001, and the iteration number is 5,000. The captured data samples are 410 samples, with 330 training samples, 30 verification samples, and 50 test samples.

## Results

**FIGURE 5:**
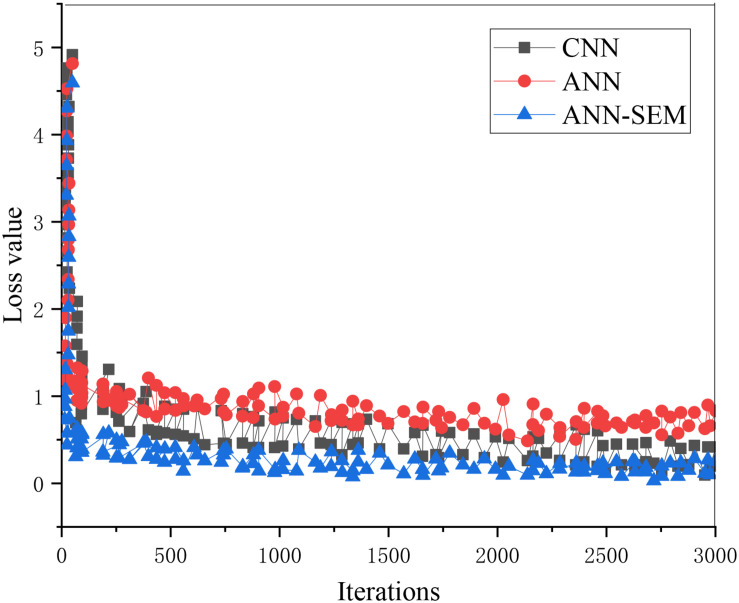
Trend graph of parameter loss values in the training set.

**FIGURE 6:**
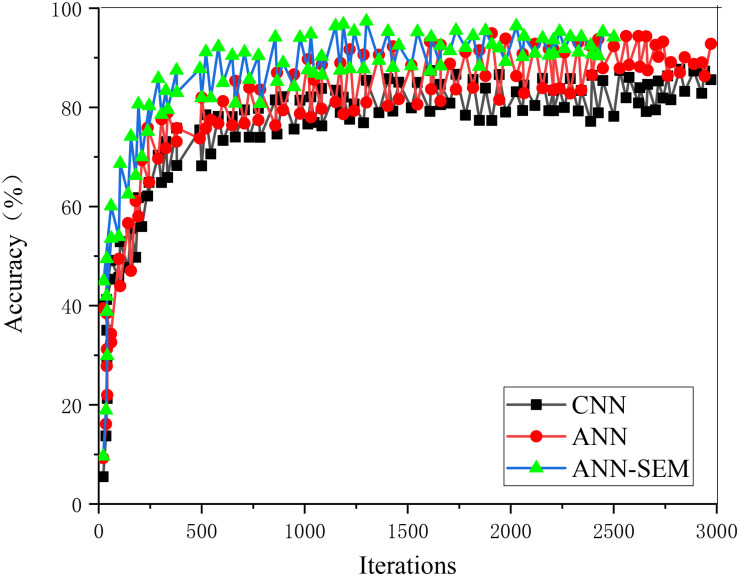
Trend graph of parameter accuracy in the training set.

The loss value and accuracy of the ANN, CNN, and hybrid network of ANN and CNN during model training are analyzed and compared, obtaining the results of Figures 5, 6.

**FIGURE 7:**
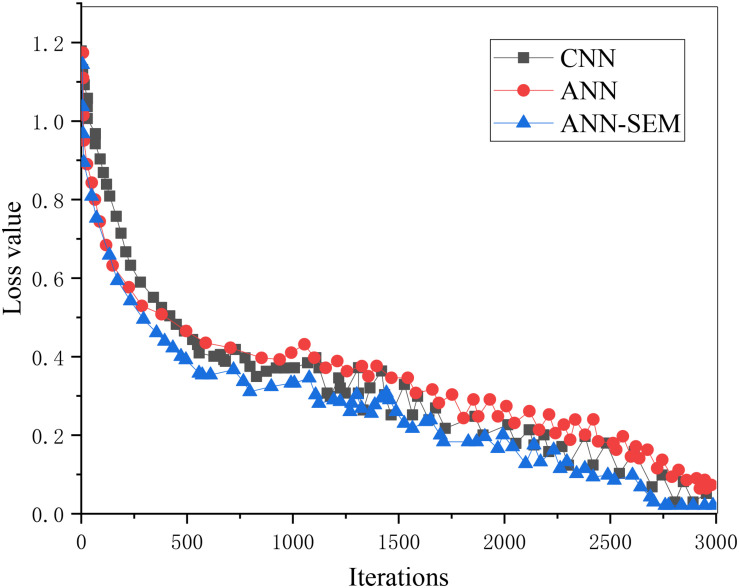
Trend graph of parameter loss values in the test set.

**FIGURE 8:**
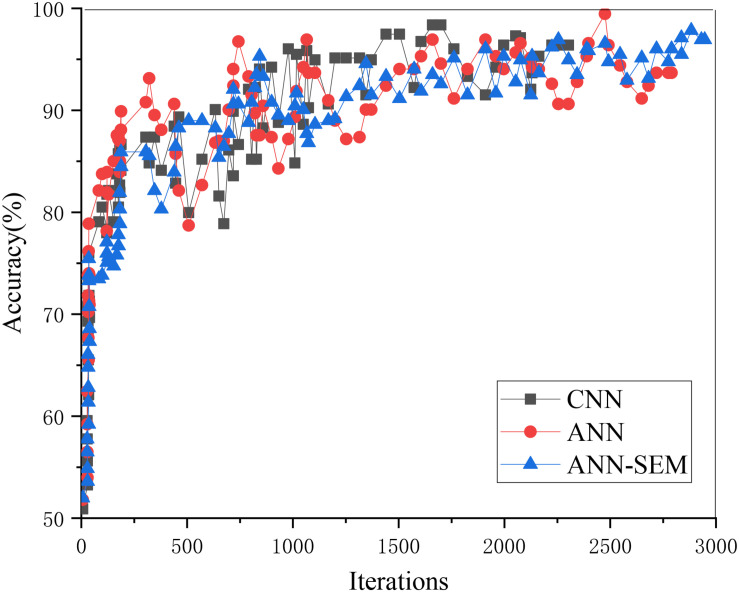
Trend graph of parameter accuracy in the test set.

Figures 5, 6 show that as the iteration number increases, the three network models’ loss values during training parameters are gradually decreasing. The hybrid network’s loss value based on ANN and CNN gradually approaches 0.15, and its accuracy approaches 96%. The loss value and accuracy during the test are shown in Figures 7, 8.

The above two figures indicate that when the iteration number reaches 3000, its accuracy reaches more than 85%. The reason is that the data obtained through the crawler may cause deviation in the processing process, which, in turn, impacts accuracy. Overall, the test results have almost met expectations.

Hence, the results of the precision, recall, and F-Score of these three methods are obtained, as shown in Table 4.

**TABLE 4:** Model evaluation results.

Model	*P*-value	*R*-value	*F*-value
CNN	89.1	87.2	88.7
ANN	90.1	88.5	90.3
ANN-SEM	95.1	94.7	94.8

The above results show that the ANN-SEM hybrid algorithm’s information classification performance is better than that of the pure neural network, and its accuracy is improved by 6 and 5%, respectively.

When training the model, its iteration number and accuracy are shown in Figure 8.

**FIGURE 9:**
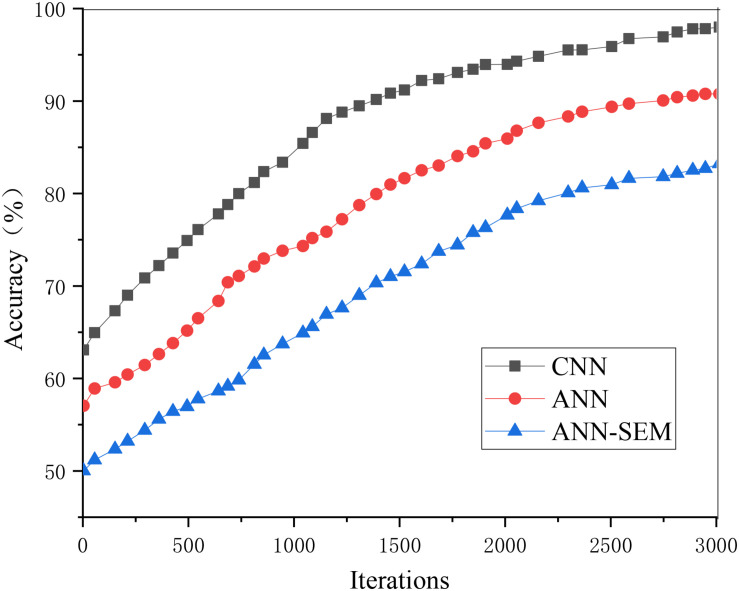
Iterative results of the prediction model.

Figure 9 shows that the hybrid network’s accuracy tends to be stable when iterating about 3,000 times, and the corresponding accuracy is higher than that of the pure neural network.

**FIGURE 10:**
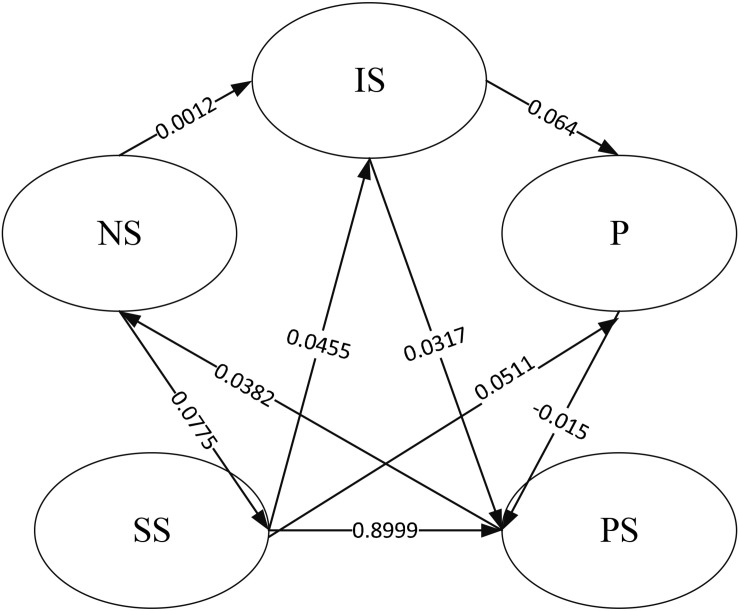
Path coefficient output results.

The output results of the SEM’s path coefficients are shown in Figure 10.

The above figure indicates that the path coefficient between the prevention and the patient situation is -0.015, showing that the relationship between the two is not apparent, and the indirect positive effect is significant. The path coefficient between the school state and the patient situation is 0.8999, showing that the natural state has a more significant impact on the school situation.

**FIGURE 11:**
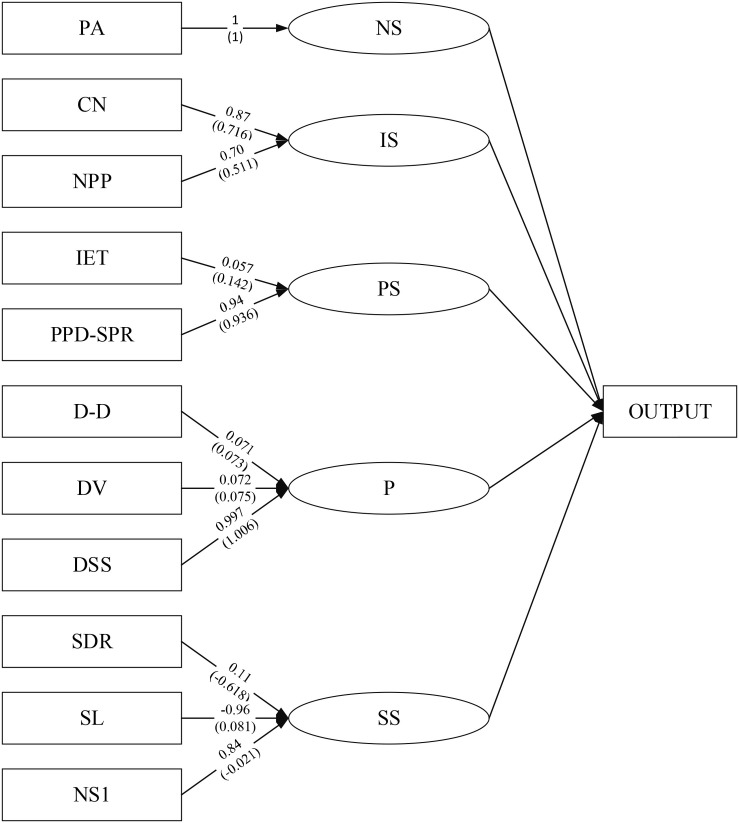
Measurement model.

The measurement model’s load and weight results are shown in Figure 11.

Figure 11 shows that consecutive case number affects the infection degree to a greater extent. The lower the school level, the greater the impact on the school state. The fewer the school’s student number, the safer the school is, and the school’s better. The higher the strong positive rate, the greater the impact on the disease state. The longer the exposure time of the infection source also affects the disease state to a certain extent. In schools, the dormitory staffing situation has a more significant impact on the prevention status. Although ventilation and disinfection have an impact, the overall impact is relatively low.

After classifying the 50 pieces of information data from June 2017 to 2019, the time in each level is selected for analysis and processing. Also, errors are analyzed. The accuracy reaches 95.1%, which shows some errors in the prediction process, but the overall classification is useful. The three models’ classification is shown in Table 5.

**TABLE 5:** Model classification results.

Prediction model	Level I threat	Level II threat	Level III threat	Level IV threat	Accuracy
ANN	11	9	13	8	89.1%
CNN	10	9	15	6	90.1%
ANN-SEM	12	10	10	5	95.1%

Among the 50 pieces of information data, there are 13 pieces of early warning data, including three pieces of Level I, four pieces of Level II, four pieces of Level 3, and 2 pieces of Level IV. This result is substituted into the numerical Equation for the warning level, and the *T-*value can be obtained, which is 6.3. *T*’s value range determines that the event is a Level IV response.

## Discussion

In deep learning technology, the ANNs’ application to construct early warning methods for public health emergencies can accurately predict public health emergencies. CNN and ANN train the model due to CNN’s advantages in image and speech recognition, thereby obtaining the loss value and prediction accuracy. The results also show that the hybrid ANN-SEM training’s loss value approaches 0.15, and its corresponding accuracy reaches 96%. In the test, the loss value decreases, and the accuracy increases. However, because crawlers realize the information data, there may be some deviations in the data processing process, impacting the accuracy. As the iteration number continues to increase, the parameter accuracy tends to be stable, and the results have almost reached expectations.

The precision, recall, and F-Score of the ANN-SEM network, CNN, and ANN are analyzed. ANN-SEM’s information classification performance is better than the other two networks, and its precision is also improved by more than 5%. The result shows that the hybrid network proposed has better classification performance.

Then, the SEM analyzes the pathological data and indicators to obtain SEM’s path coefficient. The results show that the path coefficient between the prevention and the patient situation is negative. The relationship between the two is not apparent, but there is an indirect positive effect. When there is no good prevention, although the patient situation is not directly affected, it will impact the patient situation if it affects the environment and surrounding conditions. The path coefficient between the school state and the patient situation reaches 0.8999, which shows that the influence significantly impacts the patient situation, which needs to be focused on in the subsequent prediction process. The SEM measurement model shows that the lower the school level, the fewer the school’s student number, and the better the school state. The higher the strong positive rate, the more significant the patient situation’s impact, and the longer the exposure time of the infection source will increase the patient situation. The dormitory staff setting, ventilation, and disinfection frequency will also impact the patient situation, but overall, the impact is relatively small. By controlling the student number and the strong positive rate, the patient situation can be reduced.

Finally, after classifying and analyzing the collected information data, the hybrid network’s effectiveness is further verified by comparing the three networks’ classification and accuracy. Meanwhile, among the existing information data, 13 pieces of early warning data can be accurately classified. When using these data to calculate the warning level, the final *T*-value also indicates that the event belongs to the Level IV response, and the prediction method is effective.

The information data collected from June 2017 to June 2019 is processed to meet the prediction expectations of public health emergencies so that the constructed prediction model faces fewer interference factors. It may also affect the experimental results.

When using the ANN-SEM network to classify the collected information data and analyze the loss value and accuracy of training and testing, the hybrid network can obtain higher accuracy. It provides a more reliable prediction method for SEM and can accurately determine the early warning level corresponding to the event when predicting public health events. The hybrid method based on ANN-SEM is effective in event prediction, which provides an early warning basis for the health department and provides prediction ideas for other emergencies.

## Conclusion

The deep learning technology and ANN are combined based on the SEM’s application to determine hidden and explicit variables. CNN processes and analyzes the information data obtained by the crawler. Also, ANN classifies the data, and the early warning method’s effectiveness is verified by comparing the loss value and accuracy. However, the investigation of the model’s optimized content is less in the process. Then, the model optimization will be deeply explored to improve the early warning model’s accuracy. With the information data’s continuous increase, the processing of information data becomes particularly significant. The advantages of deep learning technology and other neural network methods in image recognition and data processing provide more possibilities for related fields. Therefore, against the deep learning background, the investigation of public health emergencies’ early warning methods based on ANN is incredibly significant for judging and predicting the disease development trend. Moreover, it has a particular reference value for the investigations of other emergencies’ early warning methods.

## Data Availability Statement

The raw data supporting the conclusions of this article will be made available by the authors, without undue reservation, to any qualified researcher.

## Ethics Statement

The studies involving human participants were reviewed and approved by the NingboTech University Ethics Committee. The patients/participants provided their written informed consent to participate in this study. Written informed consent was obtained from the individual(s) for the publication of any potentially identifiable images or data included in this article.

## Author Contributions

SZ conceived the analysis question, conducted the analysis also and critically revised the manuscript content. XH was the organizer of the project and responsible for sorting out the contact data and connecting with researchers.

## Conflict of Interest

The authors declare that the research was conducted in the absence of any commercial or financial relationships that could be construed as a potential conflict of interest.
